# Programmable calculus operations in electromagnetic space using space-time-coding metasurface

**DOI:** 10.1093/nsr/nwag249

**Published:** 2026-04-30

**Authors:** Hao Tian Shi, Lei Zhang, Rui Yuan Wu, Yi Ning Zheng, Xiao Qing Chen, Yuanzhe Li, Shi He, Jun Wei Wu, Qiang Cheng, Tie Jun Cui

**Affiliations:** State Key Laboratory of Millimeter Waves, Southeast University, Nanjing 210096, China; State Key Laboratory of Millimeter Waves, Southeast University, Nanjing 210096, China; College of Information Science and Engineering, Hohai University, Nanjing 211100, China; State Key Laboratory of Millimeter Waves, Southeast University, Nanjing 210096, China; State Key Laboratory of Millimeter Waves, Southeast University, Nanjing 210096, China; State Key Laboratory of Millimeter Waves, Southeast University, Nanjing 210096, China; National Key Laboratory of Automatic Target Recognition, College of Electronic Science and Technology, National University of Defense Technology, Changsha 410073, China; State Key Laboratory of Millimeter Waves, Southeast University, Nanjing 210096, China; State Key Laboratory of Millimeter Waves, Southeast University, Nanjing 210096, China; State Key Laboratory of Millimeter Waves, Southeast University, Nanjing 210096, China; State Key Laboratory of Millimeter Waves, Southeast University, Nanjing 210096, China

**Keywords:** space-time-coding metasurface, programmable, mathematic operation, calculus operation, electromagnetic space

## Abstract

With the rapid advancement of metasurfaces and the increasing demand for programmable metasurfaces to simplify information systems, wave-based computation using metasurfaces has emerged as an attractive research topic. To facilitate the mathematical operations in electromagnetic (EM) space, here we propose a space-time-coding metasurface (STCM) system capable of directly performing calculus operations on the spatial energy distributions of EM waves. By exploiting harmonic characteristics induced by time-varying coding, the responses of meta-atoms at specific harmonics can be flexibly controlled, which enables the metasurface system to address more complex tasks. Owing to its programmability, the STCM can dynamically switch functions in real time to accommodate different calculus tasks. To fully leverage the capability of the STCM, we not only present the space-time-coding sequences for differentiation and integration of EM waves, but also develop and numerically simulate the space-time-coding sequences that can independently and simultaneously implement different calculus operations on the same incident EM waves. To experimentally validate the feasibility of the EM calculus operations, proof-of-concept experiments are conducted using a programmable 2-bit STCM. Good agreements among the theory, numerical simulations, and experiments confirm the feasibility of performing calculus operations in the EM space and demonstrate the broad application prospects of the STCM in EM wave manipulations, wireless communications, and signal processing.

## Teaser

The space-time-coding metasurface is leveraged to perform programmable calculus operations in electromagnetic space, enabling versatile applications in wireless communication, sensing, and signal processing.

## INTRODUCTION

The proposal of programmable electromagnetic (EM) metasurfaces [1] provides a flexible and powerful approach for manipulating EM waves, which can be widely used in complex EM regulation [2–6], wireless communication [7–10], and sensing [11–13]. With the development of EM information theory [14,15], using metasurfaces to directly characterize and process information has become an appealing topic, one of the key challenges of which is the direct implementation of mathematical operations on spatial EM waves using programmable metasurfaces. Compared with digital computation that relies on complex computing hardware and a large amount of storage resources, performing mathematical operations directly on spatial EM waves is an analog computing paradigm with quasi-speed-of-light processing capability [16–18], which can directly process the EM signal in physical space and reduce signal processing complexity in the digital hardware.

The convolution [19] and addition [20] operations on coding metasurfaces have provided both theoretical foundations and feasible solutions for direct computing using the EM waves. Although convolution and addition operations are widely applicable to functions such as beamforming and beam steering, traditional programmable metasurfaces are constrained by a limited number of coding states, which makes it difficult to perform more complex mathematical operations. One possible solution to overcoming this limitation is the use of stacked metasurfaces. Through careful structural design and coding patterns, these stacked metasurfaces can realize complex functionalities through diffraction, such as an all-EM-based artificial neural network [21–23], optical holography, and optical encryption [24,25], thereby enabling the approximation of various mathematical operations. To date, stacked metasurfaces have been explored in diverse applications, including direction of arrival (DOA) estimation [26], computational image reconstruction [27], and intelligent sensing [28]. However, programmable stacked metasurfaces suffer from inherent drawbacks, such as the increased system profile and complicated control architectures. Another possible solution to the limited coding states is the use of passive metasurfaces with fixed physical configurations. The phase and amplitude responses of a passive metasurface can be flexibly engineered through the geometric structure of meta-atoms, and recent studies have shown experimentally complex functionalities such as metasurface-based intelligent systems [21–23,29], solving linear equations [30], and performing calculus operations [31,32] on spatial EM waves. Nevertheless, once fabricated, the function of a passive metasurface is fixed, which restricts it to a finite category of problems, thus limiting its capability for multifunctional and adaptive operations.

One promising solution to the aforementioned limitation is the adoption of space-time-coding metasurfaces (STCMs) [33] for EM mathematical operations. By introducing temporal modulation into the coding scheme, the STCMs can precisely control EM waves through an increased degree of time complexity. With carefully designed periodic coding sequences, the amplitude and phase responses at a specific harmonic frequency can be almost continuously tuned. Owing to the space-time-coding strategy and harmonic modulation characteristic, the STCMs are well-suited for realizing a wide range of complex functionalities. Numerous studies have been conducted on the STCMs, such as coding optimization [34,35], EM manipulation [36–41], signal processing [40–44], and wireless communications [45–50], demonstrating their broad application potentials in scattering and radiation manipulation [38,42,51–54], hardware diagnostics [55,56], and imaging [57,58] in radio-frequency technology. Thus, employing the STCM provides an effective and versatile approach for implementing complex mathematical operations and reaching programmable functional features.

To verify the effectiveness of the STCM in programmable EM mathematical operations, we design a wave-based programmable calculus operation platform based on a programmable 2-bit STCM with a specially designed space-time-coding strategy. The 2-bit coding feature provides sufficient freedom for tailoring harmonic responses, while its programmability enables the multifunctional capability that is required for implementing complex functions. For the proposed STCM, coding sequences corresponding to different functions, including differential and integral operations, are optimized and preloaded in the field-programmable gate array (FPGA) module, enabling the STCM to perform various functions in response to different control instructions. Inspired by the convolution theory of metasurfaces and Fourier relationships between the near-field and far-field EM wave distribution, we carefully optimized several sets of space-time-coding sequences, which can realize differential and integral operations in the EM space. Furthermore, by exploiting the harmonic resources, we design a space-time-coding sequence that can perform the differential operation at the first harmonic and integral operation at the second harmonic simultaneously for the same incident wave. Good agreement between the design objectives and measured results demonstrates satisfactory performance of the STCM platform in implementing EM mathematical operations, highlighting its broad application potential in EM manipulation, signal processing, and imaging. In contrast to a previous study on metasurface-based mathematical operations which relied solely on one harmonic frequency [59] and only focused on theoretical analysis and numerical simulations, this work not only realizes a realistic STCM prototype for programmable EM calculus operations but also fully exploits multi-harmonic resources to enhance functionality.

## FRAMEWORK OF THE METASURFACE-BASED CALCULUS PLATFORM

**Figure 1.**
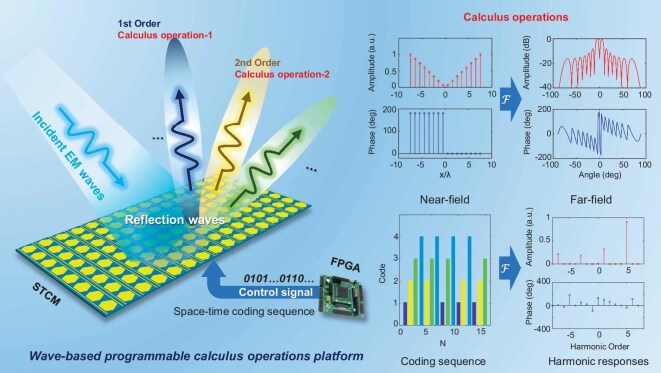
Schematic illustration of the wave-based programmable calculus operations platform that can conduct different calculus operations in the +1st and +2nd harmonics using the STCM. The STCM is controlled by a FPGA, and can characterize the calculus operators through different space-time-coding sequences and realize direct calculus operations towards the incident EM wave.

Figure 1 illustrates the structural design of the proposed STCM-based calculus platform, along with a schematic representation of implementing differential operators. The STCM calculus platform is operated by exploiting two Fourier transform relationships, namely the Fourier relationships between the time-coding sequences and frequency-domain harmonics, and the Fourier relationships between the near-field responses and far-field scattering patterns. By leveraging these two Fourier transform mechanisms, calculus operations on incident EM waves can be effectively realized.

The Fourier transform relation between the time-coding sequence and harmonic response provides a promising solution for the arbitrary manipulations of the amplitude and phase responses at a specific harmonic frequency, thereby enabling the characterization of calculus operators on a metasurface. For a time-coding sequence of a given length *L*, the relationship between the coding sequence *c_n_* and the harmonic response *Γ_k_* can be expressed as [26]:


(1) \begin{eqnarray*}
{\Gamma }_k = \mathop \sum \limits_{n = 1}^L {c}_n{e}^{ - j\frac{{k2\pi n}}{L}}.
\end{eqnarray*}

The Fourier transform relation between the near-field EM response and the far-field pattern enables the representation of different mathematical operators implemented by the proposed metasurface, thereby allowing calculus operations to be performed in the far-field scattering pattern. For a metasurface with continuous electromagnetic response, the near-field response *r(x)* and the corresponding one-dimensional far-field scattering pattern *R(θ)* can be represented as:


(2) \begin{eqnarray*}
R\left( \theta \right) = \mathop \smallint \limits_{{x}_{min}}^{{x}_{max}} r\left( x \right){e}^{ - j\frac{{2\pi x\sin \theta }}{\lambda }}{\rm d}x,
\end{eqnarray*}

where λ is the wavelength of the incident EM waves. For the proposed metasurface adopted in this work, the EM response is discretized in space. Accordingly, the discrete near-field response *r(x_i_*) and the scattering pattern *R(θ)* can be further represented as:


(3) \begin{eqnarray*}
R\left( \theta \right) = \mathop \sum \limits_{i = 1}^N r\left( {{x}_i} \right){e}^{ - j\frac{{2\pi {x}_i\sin \theta }}{\lambda }},
\end{eqnarray*}

where *x_i_* is the center of the meta-atom, and *N* is the number of meta-atoms.

When conducting the mathematical calculations, the proposed STCM is illuminated by a monochromatic EM wave that generates a near-field distribution *f(x)* according to the far-field distribution *F(θ)*. The metasurface generates a phase and amplitude distribution *r(x_i_*) at a specific harmonic to represent a mathematical operator. The output of the mathematical calculation *F*_1_*(θ)* can be obtained by measuring the resulting far-field pattern, where *F*_1_*(θ)* can be expressed as:


(4) \begin{eqnarray*}
{F}_1( \theta ) = \mathop \sum \limits_{i = 1}^N f\left( {{x}_i} \right)r( {{x}_i}){e}^{ - j\frac{{2\pi {x}_i\sin \theta }}{\lambda }}.
\end{eqnarray*}

Owing to the Fourier transform relation, the multiplication of *f(x)* and *r(x_i_*) in the near-field corresponds to the convolution of *F(θ)* and *R(θ)* in the far field. Therefore, if the phase and amplitude distribution *r(x_i_*) is optimized by a calculus operator, the metasurface can perform the calculus operations for the incident EM waves in the far-field scattering pattern.

## THEORETICAL RESULTS AND NUMERICAL SIMULATIONS

**Figure 2.**
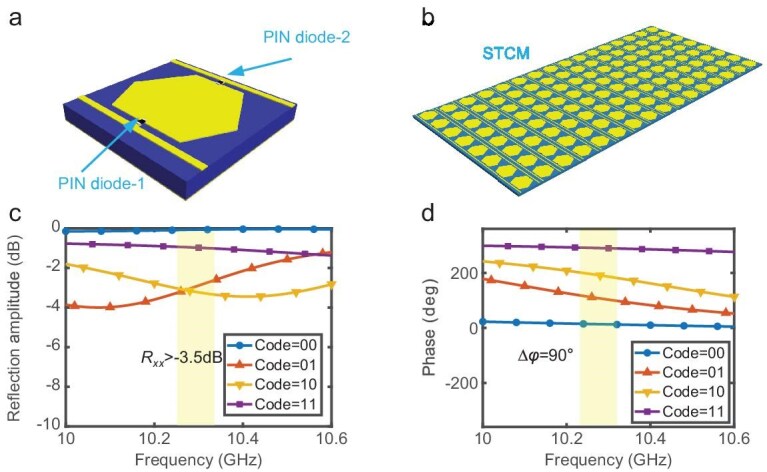
Structure and reflection responses of the STCM meta-atom. (a) Structure of the meta-atom. (b) Schematic view of the realized 2-bit STCM prototype with 16 columns. (c) Reflection amplitudes of the meta-atom with different coding states. (d) Reflection phases of the meta-atom with different coding states.

To implement practical STCM-based calculus operations, we adopt a 2-bit programmable metasurface as the hardware platform. The configuration of the employed metasurface and its EM reflection response are shown in Fig. 2, where the highlighted region is the frequency range (10.27 GHz–10.33 GHz) with a stable phase gradient (90°±15°) and high reflection coefficient (*R_xx_* > −3.5 dB). The detailed structure of the meta-atom can be found in our previous work [46]. The reflection response of the meta-atom is controlled by two PIN diodes that are embedded in the meta-atom. For convenience, the four different combinations of PIN-diode states are defined as four distinct binary coding states from 00 to 11. The coding states and the measured reflection responses of the meta-atom at 10.3 GHz are summarized in Table 1.

**Table 1.** Coding states and the reflection responses of the meta-atom (measured at 10.3 GHz).

Coding states	PIN diode-1	PIN diode-2	Amplitude (dB)	Phase (°)
00	OFF	OFF	−0.06	12°
01	ON	OFF	−2.86	102°
10	OFF	ON	−3.25	186°
11	ON	ON	−0.98	289°

To further evaluate the performance of the meta-atom, the measured results of the adopted meta-atom in the frequency band 10∼10.6 GHz are shown in Fig. 2c and d. These results demonstrate that the proposed meta-atom has four different phase responses with an ∼90° phase increment, and a stable reflection amplitude higher than −3.5 dB in four different coding statuses from 10∼10.6 GHz. The 2-bit coding performance confirms that the proposed metasurface can serve as an ideal hardware platform for implementing calculus operations.

To verify the concept of the STCM-based calculus platform, we conduct simulations on a prototype with 16 columns that are controlled independently, as shown in Fig. 2b. Based on the Fourier transform relation between the time-coding sequences and harmonic responses, the time-coding sequences can be obtained by inverse Fourier transform of a given harmonic response sequence if the amplitude and phase responses of the meta-atom can be manipulated arbitrarily. However, for the given 2-bit programmable metasurface, the meta-atom only has four limited reflection responses. Hence, for a given periodic time-coding sequence of length *L*, the optimization of the time-coding sequence can be regarded as a search problem in a finite state space. To accelerate the solution of the optimal time-coding sequence, a genetic algorithm (GA) is employed for the optimization process.

To show the effectiveness of GA in optimizing coding sequences, we conduct the differential and integral operations at the first-order harmonic, respectively. Since the inter-element coupling between adjacent meta-atoms is small according to previous research [46], to simplify the optimization process, we assume the meta-atoms in each column have no inter-element coupling. When conducting the differential operation in the first-order harmonic, the constraint condition *err_k_* for the space-time-coding sequence of different coding elements in the STCM with *L* independent controllable coding elements is expressed as


**Figure 3.**
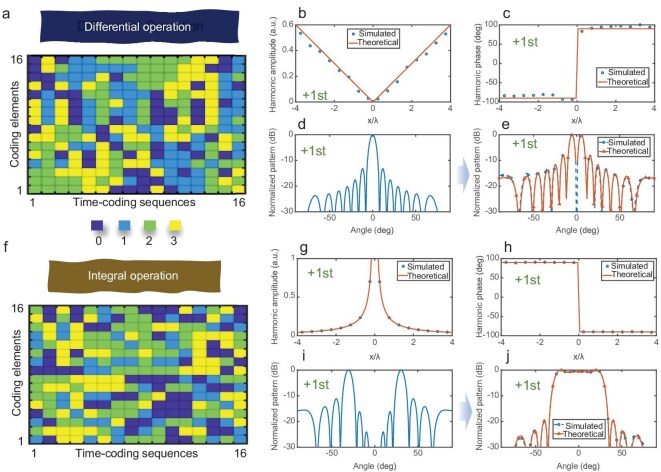
Simulated results of performing differential or integral operations at the +1st harmonic. (a) Space-time-coding sequences of the STCM for differential operation at the +1st harmonic. (b) Amplitude responses of different coding elements in the differential operation. (c) Phase responses of different coding elements in the differential operation. (d) Normalized scattering pattern of the metal plate with the same area illuminated by a normally incident plane wave. (e) Normalized scattering pattern of the metasurface illuminated by the normally incident plane wave when conducting the differential operation. (f) Space-time-coding sequences of the STCM for integral operation. (g) Amplitude responses of different coding elements in the integral operation. (h) Phase responses of different coding elements in the integral operation. (i) Normalized scattering pattern of the metal plate with the same area illuminated by differential incident waves at ±30°. (j) Normalized scattering pattern of the metasurface illuminated by differential incident waves at ±30° when conducting the integral operation.

(5) \begin{eqnarray*}
er{r}_k = \left| {\left( {k - \frac{{N + 1}}{2}} \right)jA - \mathop \sum \limits_{n = 1}^L {c}_{kn}{e}^{ - j\frac{{2n\pi }}{L}}} \right|,
\end{eqnarray*}

where *k* is the number of the coding element, and *A* is a constant value for controlling the harmonic amplitude. When *A* is specified as 0.0875 and *err_k_* is minimized, the optimization results of the STCM with 16 coding columns are presented in Fig. 3a, and the near-field harmonic responses of each meta-atom are given in Fig. 3c and d. To illustrate the performance of the differential operation, we simulate the scattering pattern of the proposed metasurface illuminated by normally incident EM waves. Figure 3d shows the scattering pattern of a metal plate with the same area as the STCM calculus platform, while Fig. 3e presents the simulation results of the STCM calculus platform performing the differential operation, alongside the theoretical differential results for the incident wave reflected from a metal plate of identical size.

For the integral operation, the constraint condition *err_k_* for the space-time-coding sequence is defined as


(6) \begin{eqnarray*}
er{r}_k = \left| {\frac{{jA}}{{k - \frac{{N + 1}}{2}}} - \mathop \sum \limits_{n = 1}^L {c}_{kn}{e}^{ - j\frac{{2n\pi }}{L}}} \right|.
\end{eqnarray*}

When *A* is specified as 0.35 and *err_k_* is minimized, the optimization results of the metasurface with 16 coding columns are shown in Fig. 3f, and the near-field harmonic responses of meta-atom are presented in Fig. 3g and h. To illustrate the performance of the integral operation, we simulate the scattering pattern of the proposed metasurface illuminated by differential incident waves at an angle of ±30°, where the incident waves from 30° and −30° have a phase difference of 180°. Figure 3i shows the scattering pattern of the metal plate with the same area as the metasurface, and Fig. 3j shows the simulated results of the STCM calculus platform performing the integral operation and the theoretical integral results of the incident waves that are reflected by the identical size metal plate.

In the harmonic responses, a good agreement between the simulated results and the theoretical values demonstrates the effectiveness of GA in optimizing the space-time-coding sequences, and the unparalleled degree of freedom for the STCM in harmonic manipulations. In the far-field scattering patterns, the STCM calculus platform can successfully split a single beam through differential operation and convert differential incident waves into a fan-shaped beam through the integral operation. When conducting the differential operation, the peaks of the reflection pattern in the simulation results are at ±5°, which are the same as the theoretical results. When conducting the integral operation, the −3 dB reflection region in the simulation results is from −28° to 28°, which are the same as the theoretical results. The good consistency between theoretical differential and integral beams and the far-field scattering pattern of the STCM demonstrates the feasibility of directly performing calculus operations on spatial EM waves using metasurfaces. These results highlight the broad application prospects for metasurfaces in EM manipulation and signal processing.

Noting that the spectrum utilization of the STCM is low and the bandwidth is limited when only one harmonic is adopted, it is essential to develop frequency multiplexing in the STCM to utilize different harmonics for different functions. To fully utilize the EM manipulation capability of the STCM and broaden its potential in EM calculus operations, a space-time-coding sequence is optimized to simultaneously perform the differential operation at the first harmonic and the integral operation at the second harmonic. The corresponding constraint condition *err_k_* for this space-time-coding sequence is defined as


\begin{eqnarray*}
er{r}_k = \sqrt {{{\left| {\left( {k - \frac{{N + 1}}{2}} \right)j{A}_1 - \mathop \sum \limits_{n = 1}^L {c}_{kn}{e}^{ - j\frac{{2n\pi }}{L}}} \right|}}^2 + {{\left| {\frac{{j{A}_2}}{{k - \frac{{N + 1}}{2}}} - \mathop \sum \limits_{n = 1}^L {c}_{kn}{e}^{ - j\frac{{4n\pi }}{L}}} \right|}}^2} . (7)
\end{eqnarray*}

To ensure the feasibility of the coding optimization, the following constraints are imposed on *A*_1_ and *A*_2_:


(8) \begin{eqnarray*}
\sqrt {{{\left[ {\left( {k - \frac{{N + 1}}{2}} \right){A}_1} \right]}}^2 + {{\left( {\frac{{j{A}_2}}{{k - \frac{{N + 1}}{2}}}} \right)}}^2} < 1.
\end{eqnarray*}

**Figure 4.**
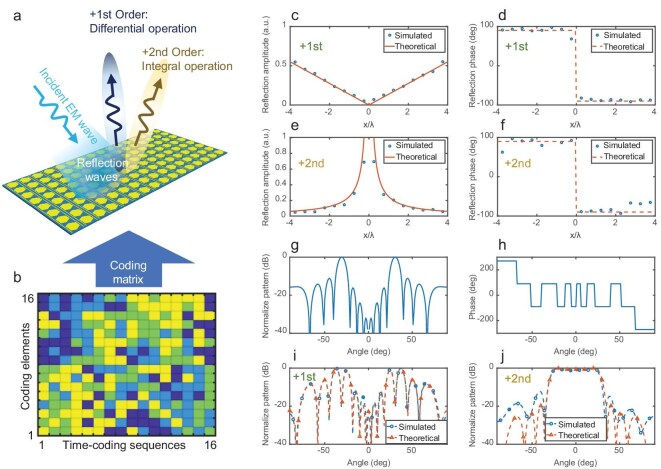
Simulated results of simultaneously performing differential and integral operations at the +1st and +2nd harmonics, respectively. (a) Schematic diagram of a STCM that simultaneously performs the differential and integral operations. (b) Space-time-coding sequences for performing the calculus operations at two harmonics. (c) Amplitude responses of different coding elements at the +1st harmonic. (d) Phase responses of different coding elements at the +1st harmonic. (e) Amplitude responses of different coding elements at the +2nd harmonic. (f) Phase responses of different coding elements at the +2nd harmonic. (g) Normalized scattering pattern of the metal plate with the same area illuminated by differential incident waves at ±30°. (h) Phase distribution of the metal plate with the same illumination. (i) Normalized scattering pattern of the STCM at the +1st harmonic with the same illumination when conducting the differential operation. (j) Normalized scattering pattern of the STCM at the +2nd harmonic with the same illumination when conducting the integral operation.

When *A*_1_ = 0.075 and *A*_2_ = 0.35, the simulated results of the STCM that simultaneously conducts the differential and integral operations in the first and second harmonics are presented in Fig. 4. Compared with the results that separately implement the differential and integral operations in the first harmonic (see Fig. 3), the amplitude and phase responses of the first and second harmonics for simultaneously differential and integral operations in Fig. 4c–f exhibit larger deviations from the theoretical values. However, the simulated results still verify the feasibility of conducting different calculus operations in different harmonics without increasing the encoding length. Moreover, the simulation points with relatively large errors typically correspond to small amplitudes, reducing their impact on the calculus operations.

To fully illustrate the performance of the STCM calculus platform, numerical simulations are conducted on scattering patterns of the first and second harmonics when the metasurface with the predesigned space-time-coding sequence is illuminated by differential incident waves at ±30°. For comparison, the reflection amplitude and phase of a metal plate with the same area under the same illumination are shown in Fig. 4g and h, while the far-field scattering patterns of the first and second harmonics are presented in Fig. 4i and j. When conducting the differential operation, the peaks of the reflection pattern in the simulation results are at ±25° and ±37°, which are the same as the theoretical results. When conducting the integral operation, the −3 dB reflection region in the simulation results is from −28° to 28°, which are the same as the theoretical results. Despite errors between simulation and theoretical calculation results in the region with a reflection pattern < −10 dB, the simulation results are in good consistency with the theoretical value, strongly supporting the application prospects of the STCM for multi-functional calculus operations.

Furthermore, to quantitatively analyze the effect of the STCM in calculus operations, parameters including energy efficiency, spectrum utilization, and normalized root mean square error (RMSE) of both simulation and theoretical results are calculated and listed in Table 2. When conducting mono-functional operation at the 1st harmonic, the efficiency and spectrum utilization of the metasurface-based calculus platform are low due to the spurious emissions in the harmonic spectrum. By multiplexing different functions in different harmonics, both efficiency and spectrum utilization can be increased at the cost of increasing the RMSE.

**Table 2.** Quantitative indicators of the simulation results.

Functionality	Differential efficiency	Integral efficiency	Spectrum utilization	RMSE in differential	RMSE in integral
Differential operation at the 1st harmonic	16.58%	–	6.25%	2.22%	–
Integral operation at the 1st harmonic	–	12.22%	6.25%	–	1.10%
Multiplexing the 1st and 2nd harmonics	15.59%	11.49%	12.5%	4.02%	1.72%

Limited by the coding length and limited coding states of the STCM, the difference between the harmonic response and the ideal response is difficult to precisely control, especially when the harmonic amplitude is high, resulting in the RMSE in the simulation. Although extending the coding length of the STCM can effectively reduce the RMSE, spectrum utilization will decrease with the extension of the coding length.

To further demonstrate the potential of the STCM to directly perform calculus operations on EM waves, we consider a practical application for image processing, where performing the differentiation on an image can enhance its edges, thereby facilitating edge detection. To demonstrate edge enhancement using the STCM calculus platform, numerical simulations are conducted with a STCM consisting of 16 × 16 independently controllable meta-atoms, in which a space-time-coding sequence with a period of 16 time slots is employed. During coding optimization, the constraint condition *err_kl_* for the space-time-coding sequence is defined as


(9) \begin{eqnarray*}
er{r}_{kl} &=& \left| \left( {\sqrt {{k}^2 + {l}^2} - \frac{{N + 1}}{2}} \right)\right.\\
&&\times \left.{e}^{ - j{{\tan }}^{ - 1}\left( {\frac{{k - \frac{{N + 1}}{2}}}{{l - \frac{{N + 1}}{2}}}} \right)}A - \mathop \sum \limits_{n = 1}^L {c}_{kln}{e}^{ - j\frac{{2n\pi }}{L}} \right|,\quad
\end{eqnarray*}

where *k* and *l* are the row and column numbers corresponding to the meta-atom.

**Figure 5.**
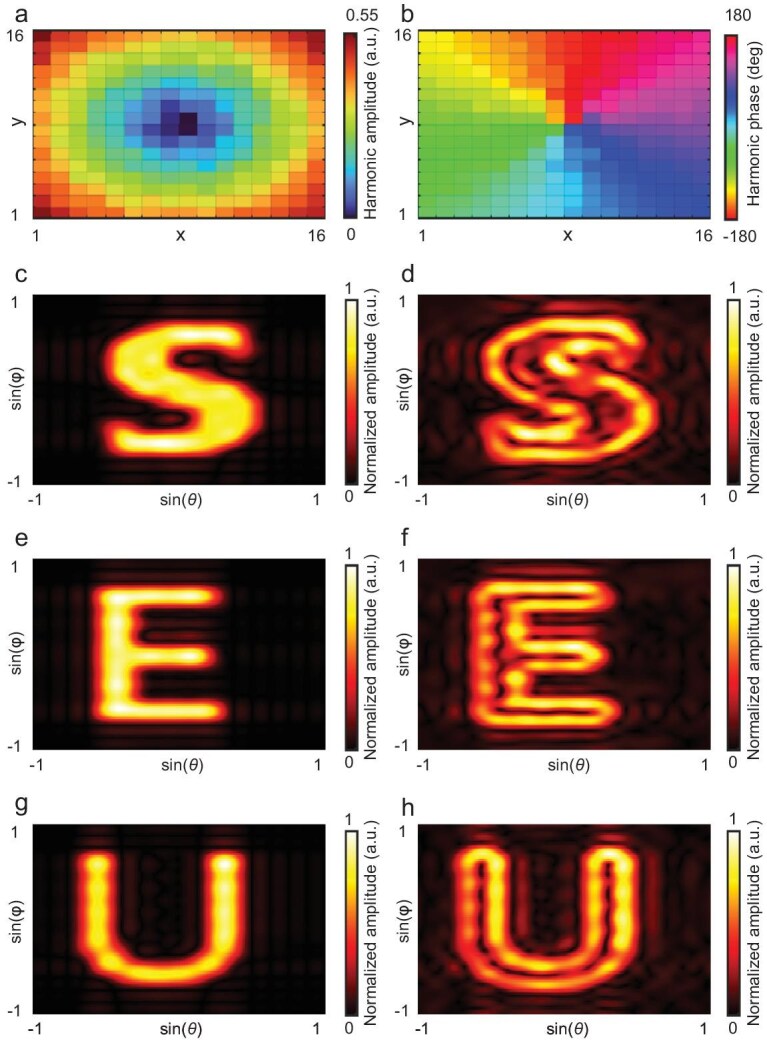
Simulated results of two-dimensional differential operations for edge enhancement at the +1st harmonic. (a) Amplitude distribution of the STCM with 16 × 16 meta-atoms. (b) Phase distribution of the STCM with 16 × 16 meta-atoms. (c–h) Scattering patterns of the metal plate (c, e, g) and STCM calculus platform (d, f, h) when conducting the edge enhancement of ‘S’, ‘E’, and ‘U’, respectively.

The simulated results of the amplitude and phase responses for the optimized space-time-coding sequence with *A*_max_ = 0.052 at the first harmonic are shown in Fig. 5a and b. These results indicate that the reflection amplitude forms a conical distribution and the phase forms a vortex distribution at the first harmonic, corresponding to the Fourier transform of a two-dimensional (2D) first-order differential operator. To verify the edge enhancement of the STCM-based calculus platform, simulations are conducted with three different structured EM waves, which form three different images, including ‘S’, ‘E’, and ‘U’, in the far-field scattering patterns of a PEC plate with the same area as the metasurface, as shown in Fig. 5c, e, and g. The corresponding simulation results of the structured EM incident waves reflected by the STCM performing 2D differential operations are shown in Fig. 5d, f, and h. In the simulation, the pitch and azimuth angles are normalized to the range of −1 to 1 through a sine function. The results demonstrate that a metasurface with 16 × 16 meta-atoms can successfully implement a 2D differential operation towards incident waves and realize edge enhancement, indicating the significant potential of the STCM-based calculus platform for image-processing applications.

## EXPERIMENTAL VALIDATIONS

**Figure 6.**
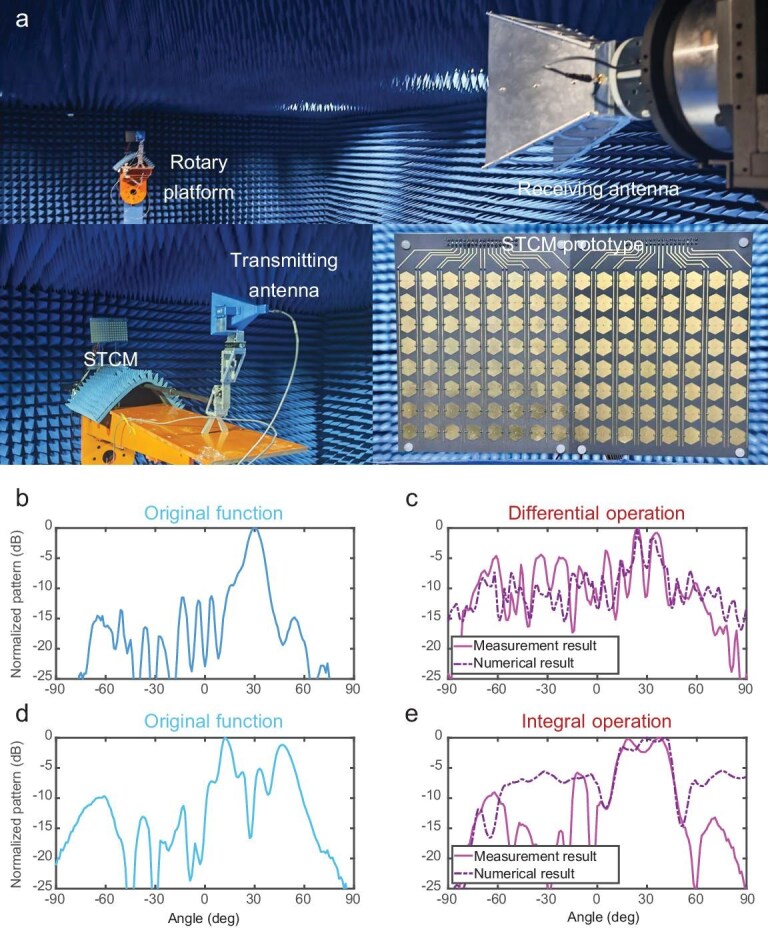
Experimental setup and measurement results of the STCM-based calculus operations. (a) Experimental setup and measurement environment of the STCM-based calculus platform. (b) Scattering pattern of the original function for the differential operation. (c) Measured and numerical calculation results of the differential operations with the original function 1. (d) Scattering pattern of the original function for the integral operation. (e) Measured and numerical calculation results of the integral operations with the original function 2.

The simulated results verify the feasibility of performing the calculus operations using STCMs. To realize a practical STCM prototype for constructing a calculus platform and evaluate its performance, we fabricated a 16 × 8 reflection-type programmable metasurface using a printed circuit board (PCB). The prototype was measured in a far-field microwave anechoic chamber to verify the function and performance, with the measurement setup shown in Fig. 6a. To enable harmonic manipulations, a 16-bit length time coding sequence with a modulation frequency of 1 MHz is generated by the FPGA to control the programmable metasurface. Since the switching speed of the coding sequence is up to 16 MHz, which is far lower than the maximum switching speed of the PIN diodes (MADP-000907–14020x) in the metasurface, errors due to the transient response of PIN diodes can be effectively reduced. To generate a monochromatic incident EM wave at 10.3 GHz, a signal generator connected to a transmitting antenna is used as the excitation source. The far-field scattering pattern is captured by a receiving antenna while the rotary platform rotates from −90° to 90°.

In the experiment, limited by the STCM platform, the metasurface can only verify 1D differential and integral operations. Furthermore, to avoid blockage of the transmitting antenna, a phase gradient is incorporated into the optimization of the space-time-coding sequence in both integral and differential operation, resulting in the reflection beam being deflected by 30°.

In the differential operation, the original function is set as a plane wave that is deflected by 30°. Since the generation of the original function through incident EM waves is difficult, both the original function and the differential result are realized by the STCM using different coding sequences. When measuring the reflection pattern of the original function, the STCM needs to characterize the response of the original function in the transform domain and, therefore, the constraint condition *err_k0_* for the space-time-coding sequence for column *k* in the original function is defined as


(10) \begin{eqnarray*}
er{r}_k = \left| {{e}^{ - j2\pi \frac{{kd}}{\lambda }\sin \frac{\pi }{6}} - \mathop \sum \limits_{n = 1}^L {c}_{kn}{e}^{ - j\frac{{2n\pi }}{L}}} \right|,
\end{eqnarray*}

where *d* is the width of each column, and *λ* is the operation wavelength. For the differential operation, the STCM needs to characterize the result of multiplying the original function by the differential operator in the transform domain and, therefore, the constraint condition *err’_k_*_1_ for the space-time-coding sequence for column *k* is defined as


(11) \begin{eqnarray*}
er{r}^{\prime}_k &=& \left| \left( {k - \frac{{N + 1}}{2}} \right)\right.\\
&&\left.\times j{A}_{max}{e}^{ - j2\pi \frac{{kd}}{\lambda }\sin \frac{\pi }{6}} - \mathop \sum \limits_{n = 1}^L {c}_{kn}{e}^{ - j\frac{{2n\pi }}{L}} \right|.\\
\end{eqnarray*}

Theoretically, the far-field scattering pattern of the original function at the first harmonic forms a single beam pointing at 30°, whereas the scattering pattern of the STCM performing the differential operation exhibits a split beam with a null at 30° and two peaks located at the rising and falling edges of the original function. To evaluate the performance of the differential operation, the numerical differential result of the original function 1 and the measured result of the scattering pattern of the STCM implementing the differential operation are shown in Fig. 6c. The peaks of the reflection pattern in the differential operation are located at 24° and 36°, which are close to those in the numerical differential result, 25° and 35°. The null steering location in the measurement result is the same as the numerical differential result. The quantitative results indicate that the position of the two peaks and the null steering in the measurement results are consistent with the numerical differential result, and the RMSE between the numerical results and measurement results in the whole reflection space is 10.52%, which is acceptable. Although discrepancies are observed in the sidelobe region, both numerical and measured results present excellent differential performance in the main-lobe region, showing fair agreement with the theoretical predictions.

For the integral operation, since generating a differential incident wave is challenging, a method similar to the differential operation is adopted, where the original function consists of a differential beam with 30° deflection and the differential results are realized by the STCM using different coding sequences. To form a pair of beams with a phase difference of 180°, a 1-bit phase coding sequence *C*_0_ is employed to constrain the harmonic response of the metasurface element, where *C*_0_ = 0000111100001111. A phase gradient capable of deflecting reflection beams by 30° is incorporated into the optimization of the space-time-coding sequence in the original function and integral operation. Thus, to characterize the original function in the transform domain, the constraint condition *err_k_* for optimizing the coding sequence for the original function is defined as


(12) \begin{eqnarray*}
er{r}_k = \left| {{e}^{ - j\left( {2\pi \frac{{kd}}{\lambda }\sin \frac{\pi }{6} - {C}_0\left( k \right)\pi } \right)} - \mathop \sum \limits_{n = 1}^L {c}_{kn}{e}^{ - j\frac{{2n\pi }}{L}}} \right|.
\end{eqnarray*}

To realize integral operation, the coding sequence is optimized to characterize the multiplication of the original function by the differential operator in the transform domain, where the constraint condition *err’_k_*_1_ for optimizing the integral operation is defined as


(13) \begin{eqnarray*}
er{r}^{\prime}_k &=& \left| \frac{{j{A}_{max}}}{{k - \frac{{N + 1}}{2}}}{e}^{ - j\left( {2\pi \frac{{kd}}{\lambda }\sin \frac{\pi }{6} - {C}_0\left( k \right)\pi } \right)}\right. \\
&&\left.- \mathop \sum \limits_{n = 1}^L {c}_{kn}{e}^{ - j\frac{{2n\pi }}{L}} \right|.
\end{eqnarray*}

Theoretically, the scattering pattern of the original function at the first harmonic forms two symmetrical beams around 30°, while the scattering pattern of the metasurface performing the integral operation produces a beam with stable amplitude around 30°. The measurement results are presented in Fig. 6d and e. Specifically, the measured scattering pattern of the original function in the integral function case exhibits two lobes with a 180° phase difference at 13° and 47°. In contrast, the scattering pattern resulting from the integral operation maintains a stable scattering pattern with a normalized amplitude higher than −3 dB over the angular range from 13° to 43°, which is close to the −3 dB region (11°–47°) in the numerical integral results of the original function presented in Fig. 6e. The normalized RMSE between the measurement results and numerical results in the −3 dB region is 6.35%. The −3 dB region between the numerical and measurement results is in good agreement, while the RMSE within the −3 dB reflection region is relatively low. Although discrepancies between the measured and numerical results are observed in the sidelobe region, which significantly improves the normalized RMSE in the total reflection space (35.47%), the numerical integration exhibits satisfactory consistency with the measured results in the integral region, where the normalized reflection pattern is higher than −3 dB. Since the power levels in sidelobes are quite low, the results of the experimental measurement are not as precise as expected, resulting in a higher error between numerical and measurement results. Overall, the measured results around 30° show good consistency with the theoretical analysis, confirming the feasibility of implementing the integral operation using the STCM.

Limited by the column-controlled STCM platform adopted in this research and the harmonic response error, the 2D calculus operations and simultaneous performing of the integral and differential operations in the 1st and 2nd harmonics are difficult to realize. Though increasing the length of the space-time-coding sequence provides a possible solution to achieve the multiplexing of harmonics at a specific order and reduce the error in harmonic response, a longer coding sequence will also introduce more complex harmonic responses and reduce spectrum utilization. Limitations in the experiment can be fundamentally solved by improving the design of the metasurface. For example, a programmable metasurface with phase and amplitude regulation capability can realize precise control over the harmonic response without increasing the length of the coding sequence and, therefore, reduce the errors and improve spectrum utilization.

As a summary, despite the measurement error discrepancies arising from the unideal conduction rate of PIN-diodes, limited aperture, and the inevitable differences between the harmonic and ideal responses of the metasurface, the experimental results obtained from the STCM -based calculus platform demonstrate its powerful ability to perform calculus operations in the EM space. To improve the performance of the metasurface-based calculus operation, the following methods, including extending the time-space coding length, expanding the scale of the metasurface, establishing a more precise model for the transient response of the STCMs for coding optimization, and improving the design of the metasurface, can be adopted, thus utilizing the potential of the proposed metasurface-based calculus platform.

## CONCLUSION

We propose a programmable STCM-based calculus operation platform. A carefully designed reflection-type metasurface with a 2-bit phase response and stable reflection amplitude provides a robust hardware foundation for the EM wave-based calculus platform. By using different time-coding sequences, the reflection phase and amplitude at specific harmonics can be flexibly and precisely controlled, enabling the STCM to emulate the differential and integral operators in the Fourier transform domain. Leveraging the Fourier transform relationship between the near-field response and far-field scattering pattern, the proposed STCM realizes direct calculus operations in the EM space. Compared with previous approaches, the STCM calculus platform exhibits versatile, reprogrammable, and multifunctional operations with a low profile. Moreover, the programmable nature of the STCM offers significant potential for performance enhancement and functional extension. Good agreement among theoretical analysis, numerical simulations, and experimental measurements confirms the feasibility of STCMs to perform calculus operations in the EM space, highlighting the broad application prospects of STCMs in wireless communication, remote sensing, and signal processing.

## Supplementary Material

nwag249_Supplemental_File
