# Supplementary material for: Programmable calculus operations in electromagnetic space using space-time-coding metasurface
Source: Natl Sci Rev. 2026 Apr 30;13(10):nwag249. doi: 10.1093/nsr/nwag249 (PMC13240848; doi:10.1093/nsr/nwag249)
Supplement: nwag249_Supplemental_File [file nwag249_supplemental_file.pdf]

Supplementary Materials for

Programmable calculus operations in electromagnetic space using space-time-coding metasurface

Hao Tian Shi[1,†], Lei Zhang[1,†,*], Rui Yuan Wu[2,*], Yi Ning Zheng[1], Xiao Qing Chen[1], Yuanzhe Li[1,3], Shi He[1], Jun Wei Wu[1], Qiang Cheng[1], and Tie Jun Cui[1,*]

[1] *State Key Laboratory of Millimeter Waves, Southeast University, Nanjing 210096, China*

[2] *College of Information Science and Engineering, Hohai University, Nanjing 211100, China*

[3] *National Key Laboratory of Automatic Target Recognition, College of Electronic Science and Technology, National University of Defense Technology, Changsha 410073, China*

[†] *These authors contributed equally to this work.*

* Corresponding authors: Email: lzseu@seu.edu.cn; ruiyuanwu@126.com; tjcui@seu.edu.cn.

This file includes:

1. GA settings and optimization details
2. Discrepancies caused by the transient response of the STCM
3. The relationship between the length of the coding sequence and the harmonic response.

1. GA settings and optimization details

Genetic algorithm (GA) is employed for the optimization of the space-time coding sequences. As a metaheuristic optimization and search technique inspired by the principles of natural evolution, GA has excellent global search capabilities[1]. Currently, GA and its derivative algorithms demonstrate outstanding performance in metasurface coding optimization[2]. When conducting GA in coding sequence optimization for the first harmonic, the fitness value F is calculated by the following function:

$$F = \left|A - \sum_{n=1}^{L} c_n e^{-j\frac{2n\pi}{L}}\right| \tag{S1}$$

where c_n is the time domain reflection response of the coding sequence, and A is the optimization target. The normalized error *err* of each iteration is calculated by the following function:

$$err = \frac{F}{A} \tag{S2}$$

When conducting GA in coding sequence optimization for the first and second harmonics, the fitness value F is calculated by the following function:

$$F = \sqrt{\left|A_1 - \sum_{n=1}^{L} c_n e^{-j\frac{2n\pi}{L}}\right|^2 + \left|A_2 - \sum_{n=1}^{L} c_n e^{-j\frac{4n\pi}{L}}\right|^2} \tag{S3}$$

where A_1 and A_2 is the optimization targets for the first and second harmonic, respectively. The normalized error *err* of each iteration is calculated by the following function:

$$err = \frac{F}{\sqrt{{A_1}^2 + {A_2}^2}} \tag{S4}$$

When conducting coding optimization, the length of the coding sequence L equals 16, and the coding states within the coding sequences are chosen from the reflection response of the metasurface. In each iteration of the GA, the k individuals with the least F are chosen as the parents to generate the next generation. The termination condition in the GA is $err<0.01$ or reaching the maximum generation.

The performance of GA is dependent on the initial conditions and parameter settings. To directly show the setting of GA and the optimization details, the flow chart of the GA and the error curve of each iteration under different optimization objectives are shown in **Figure S1**, and the details of parameters, including population size, number of generations, crossover rate,

and mutation rate, are shown in Table S1.

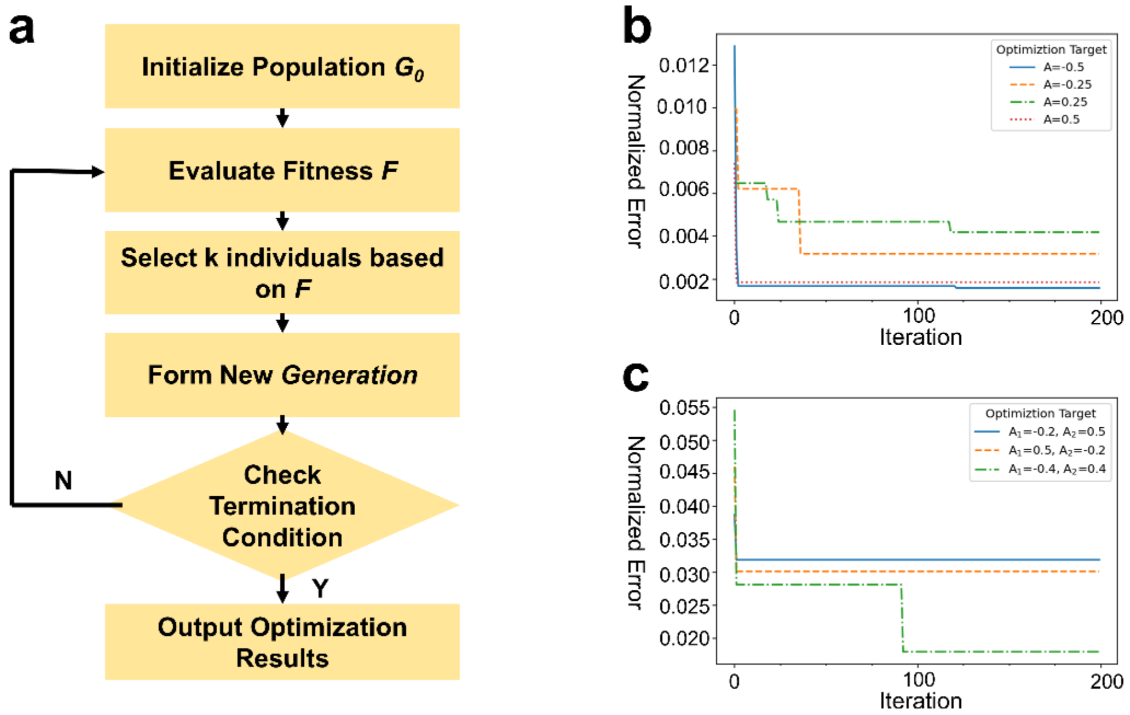

Figure S1. (a) The flow chart of the GA. (b) The normalized error of the GA in each iteration when only optimizing the responses of the first harmonic with different optimization targets in 200 iterations. (c) The normalized error of the GA in each iteration when simultaneously optimizing the responses of the first and second harmonics with different optimization targets in 200 iterations.

Table S1. Parameters in GA

Population	Generations	Crossover rate	Mutation rate
10000	200	0.5	0.05

2. Discrepancies caused by the transient response of STCM

In optimizing the coding sequence, transitions between different coding states are modeled as step responses. However, due to the non-ideal transient characteristics of the programmable metasurface during state switching, discrepancies arise between the realistic STCM reflection patterns and theoretical predictions even the measured results of the phase and amplitude of the metasurface is adopted in optimization. Although precisely modeling the transient response of STCM state switching remains challenging, the upper bound of these errors can be estimated, ensuring that deviations remain within acceptable limits with a limited switching speed. To

analyze the discrepancies caused by the transient response of the STCM, the error during the state transition is equivalent to a periodic pulse signal with the frequency of switching speed of f_s and a pulse width t_{max}.

Firstly, to analyze the error across the entire spectrum, we calculate the normalized RMSE between the ideal response F_0(t) and the realistic response F_1(t). The upper bound of the normalized power error *err* between the theoretical response $F_0(t)$ and the realistic response $F_1(t)$ can be characterized by the following formula, where A is the maximum reflection amplitude of the metasurface:

$$err = \int_0^{\frac{1}{f_s}} \frac{|F_0(t)-F_1(t)|^2}{|F_0(t)|^2} dt \leq \frac{|2*A|^2 t_{max}}{|A|^2 \frac{1}{f_s}} \tag{S5}$$

Here, we assume the worst-case scenario where the amplitude difference reaches $2A$ during t_{max}. Thus, the upper bound of the error is estimated as $4f_s t_{max}$.

To further determine how the error is transferred to the harmonics used for the calculus operation. We analyze the spectral characteristics of errors resulting from the transient response in an STCM. A periodic pulse sequence with a frequency of f_s and a width of t_{max} is adopted to qualitatively analyze this interference. The frequency response of the error err_n can be regarded as:

$$err_n = f_1 \int_{-\frac{1}{2f_1}}^{\frac{1}{2f_1}} \left(F_0(t) - F_1(t)\right) e^{-j2\pi f_n t} dt$$

$$\approx f_1 \int_{-\frac{t_{\max}}{2}}^{\frac{t_{\max}}{2}} (2A) e^{-j2\pi f_n t} dt = 2Af_s \frac{\sin(\pi f_n t_{\max})}{\pi f_n} \tag{S6}$$

The harmonics we adopt in the calculus operation is the first harmonic $f_1=f_s/16$, considering the harmonics from within $[-f_s, f_s]$, the ratio η_{err} of the total error power ERR and the error power in the first harmonic err_1 can be calculated by the following formula.

$$\eta_{err} = \frac{{err_1}^2}{ERR} = \frac{\left(2Af_s \frac{\sin(\pi f_1 t_{\max})}{\pi f_1}\right)^2}{\sum_{i=-\infty}^{\infty} \left(2Af_s \frac{\sin(\pi f_i t_{\max})}{\pi f_i}\right)^2}$$

$$< \frac{\left(2Af_s \frac{\sin(\pi f_1 t_{\max})}{\pi f_1}\right)^2}{\sum_{i=-16}^{16} \left(2Af_s \frac{\sin(\pi f_i t_{\max})}{\pi f_i}\right)^2} \tag{S7}$$

Therefore, the ratio between the power in the first harmonic, P_1, and the error power in the first harmonic P_{err1}, can be described as:

$$\frac{P_{err1}}{P_1} = \frac{ERR \times \eta_{err}}{P \times \eta} = \frac{P \times err \times \eta_{err}}{P \times \eta} = \frac{err \times \eta_{err}}{\eta} < \frac{4 f_s t_{max} \eta_{err}}{\eta} \tag{S8}$$

As a summary, when the switching period $1/f_s$ is significantly larger than the maximum switching duration t_{max}, the overall error remains small. As $f_s t_{max}$ approaches 0, the error distribution within $[-f_s, f_s]$ becomes uniform. Since both η_{err} and η are finite values, the relative error in the first harmonic also approaches 0. In this study, the switching frequency f_s is 16 MHz, and the transient duration t_{max} is estimated based on the PIN diode switching rate (approximately 2ns) [3]. Under these conditions, the discrepancies caused by the transient response of the STCM are demonstrated to be negligible.

3. The relationship between the length of the coding sequence and the harmonic response

The length of the space-time coding sequences directly affects the amplitude and phase manipulation accuracy in an STCM system. Theoretically, a longer encoding sequence implies higher encoding accuracy, but it also increases the complexity of the coding sequence design. Furthermore, a longer space-time coding sequence also means more uncontrollable high-order harmonics, thereby reducing the spectral utilization rate of the system. Hence, when determining the length of the coding sequence of the STCM, various factors such as functionality, optimization complexity, and spectral utilization need to be considered.

In our manuscript, we adopt N=16 as the coding length, which maintains sufficient harmonic response accuracy under a short coding length. To directly show the difference resulting from the coding length, we compared the performance of an STCM conducting differential operation at the first harmonic with N=8 and N=16. The simulation results are shown in **Figure S2**. From a qualitative analysis of Figure S2, the longer coding sequence obtained a more accurate harmonic response, and the RMSE between the harmonic response and the theoretical values at N=8 and N=16 is 4.88% and 2.2%, respectively.

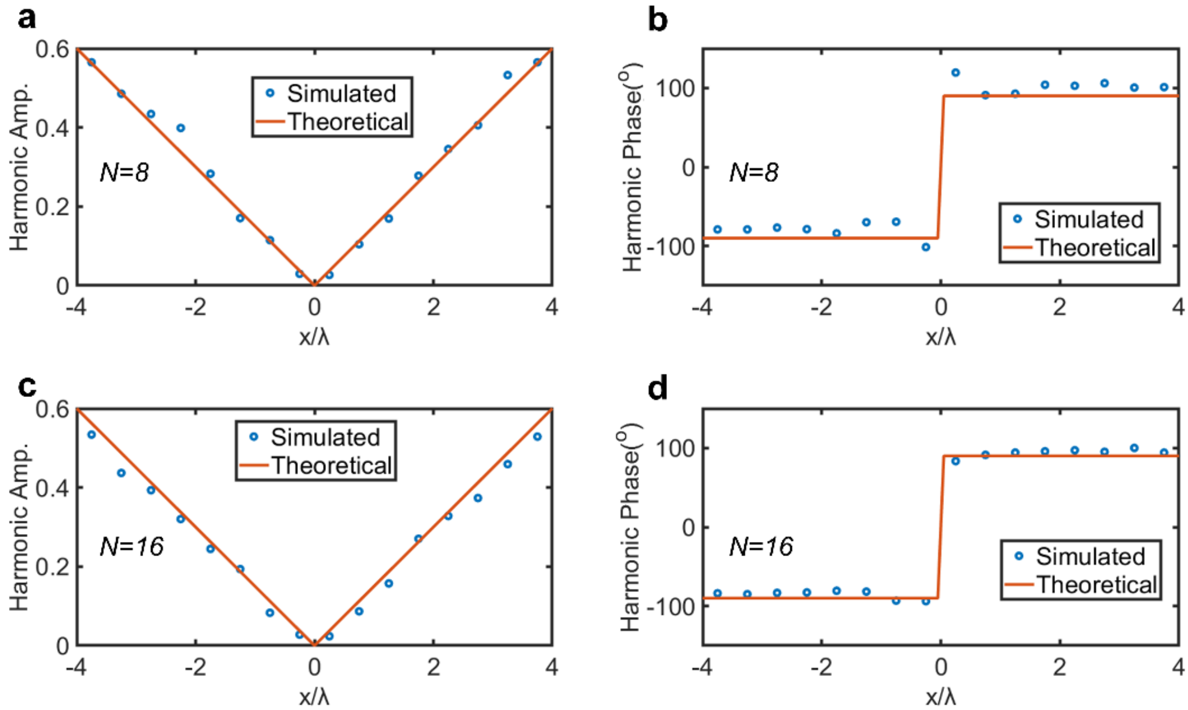

Figure S2. (a-b) The amplitude and phase responses of different coding elements in the differential operation with coding length N=8. (a-b) The amplitude and phase responses of different coding elements in the differential operation with coding length N=16.

Reference:

[1] Chatterjee S, Laudato M, Lynch LA. Genetic algorithms and their statistical applications: An introduction. Comput Stat Data An. 1996; 22(6): 633 – 651. doi: Doi 10.1016/0167-9473(96)00011-4.

[2] Yuan SSA, Jiang YT, Zhang ZY et al. Quantum Annealing-Inspired Optimization for Space-Time Coding Metasurface. Ieee T Antenn Propag. 2025; 73(9): 6512 – 6524. doi: 10.1109/Tap.2025.3573526.

[3] MACOM, MADP-000907-14020 https://cdn.macom.com/datasheets/MADP-000907-14020x.pdf.
